# Shaping ability of a pediatric motor-driven instrumentation system in primary molar root canal prototypes

**DOI:** 10.1590/0103-6440202305372

**Published:** 2023-12-22

**Authors:** Bianca Katsumata de Souza, Murilo Priori Alcalde, Marco Antonio Hungaro Duarte, Maria Aparecida Andrade Moreira Machado, Thais Marchini Oliveira, Natalino Lourenço

**Affiliations:** 1 Department of Pediatric Dentistry, Orthodontics and Community Dentistry, Discipline of Pediatric Dentistry, Bauru School of Dentistry, University of São Paulo; Bauru, Brazil; 2 Department of Operative Dentistry, Endodontics and Dental Materials, Bauru School of Dentistry, University of São Paulo; Bauru, Brazil

**Keywords:** endodontic instrumentation, primary tooth, X-Ray microtomography

## Abstract

Evaluate the shaping ability and preparation time using a pediatric motor-driven rotary instrumentation compared to other systems in resin prototypes of primary molars. Methods: Thirty specimens were scanned in micro-CT and divided into three groups according to the instrumentation type: pediatric motor-driven Sequence baby File (SBF); conventional motor-driven (Sequence Rotary File - SRF); manual K file. Instrumentation time was timed. After preparation, the specimens were scanned again. The pre- and post-instrumentation images were superimposed to measure the amount of root canal deviation and the resin remnant thickness. ANOVA followed by the Tukey test analyzed the comparisons between groups (p<0.05). Results: No statistically significant differences occurred in root canal deviation among groups (p>0.05). There were statistically significant differences in the comparison among root thirds (p<0.001) but without significant differences in the interaction group vs. root third (p>0.05). Both motor-driven instrumentations showed statistically greater weariness than manual instrumentation (p<0.001), without significant significant differences between SBF and SRF. Motor-driven instrumentation had a shorter working time than manual instrumentation (p<0.001). Conclusion: Pediatric motor-driven instrumentation demonstrated good outcomes in relation to root canal deviation and amount of remnant structure, with shorter instrumentation time. SBF can be a suitable alternative for endodontic instrumentation in primary molars.

## Resumo

Objetivo: Avaliar os resultados da instrumentação endodôntica e o tempo de preparo de lima odontopediátrica ativada por motor (Sequence Baby File- SBF) em canais de molares decíduos prototipados. Métodos: Trinta espécimes foram escaneados em micro-CT e divididos em três grupos de acordo com o tipo de instrumentação: mecanizada odontopediátrica (SBF); mecanizada convencional; lima K manual. O tempo de instrumentação foi cronometrado. Após o preparo, os espécimes foram escaneados novamente. As imagens pré e pós-instrumentação foram sobrepostas e a quantidade de desvio do canal radicular e a espessura de resina remanescente foram mensurados. Para as análises de comparações entre os grupos foram realizados teste ANOVA seguido do teste de Tukey (p<0,05). Resultados: Não ocorreram diferenças estatisticamente significativas entre os grupos no desvio do canal radicular (p>0,05). Houve diferenças estatisticamente significativas na comparação entre terços radiculares (p<0,001), mas sem diferenças significativas na interação grupo vs. terço radicular (p>0,05). A instrumentação mecanizada apresentou desgaste estatisticamente maior do que a instrumentação manual (p<0,001), sem diferenças estatísticas entre a SBF e a lima mecanizada convencional. A instrumentação mecanizada teve menor tempo de trabalho quando comparado a instrumentação manual (p<0,001). Conclusão: A instrumentação mecanizada odontopediátrica demonstrou bons resultados em relação ao desvio do canal radicular e quantidade de estrutura remanescente, com menor tempo de instrumentação. A SBF pode ser uma alternativa adequada para a instrumentação endodôntica em molares decíduos.

## Introduction

Pulpectomy is the procedure of choice in cases of irreversible pulpitis or necrosis due to the infectious process affecting the pulp, in primary teeth with little or no root resorption ^1^. This technique removes the organic debris inside the canals aiming at maintaining the tooth and periodontal health, which directly affect the function of mastication, speech, esthetics, and the child’s general development ^2^. For root canal preparation, manual or mechanized instruments can be used.

Motor-driven rotary endodontic instruments were introduced in Pediatric Dentistry in mild-2000 and from that moment on, they have been modified to improve effectiveness and working time ^3^. The literature has constantly searched for instruments with safer quality and design to avoid complications, such as flaps, deviations, and perforations, which may weaken the tooth structure or jeopardize the permanent successor ^4^. Primary molars had a complex root canal system with differences from permanent teeth: pulp chamber size and thinner dentin walls ^(^ ^5^. These features demand specific endodontic instruments for these teeth. Considering these facts, the instruments have been improved with NiTi alloys following the original curved anatomy of the canals due to the high flexibility and resistance, decreasing the instrumentation error and keeping the root morphology ^6^,^7)^. Notwithstanding, the literature lacks studies on endodontic instruments suitable for Pediatric Dentistry and on human primary teeth.

Primary teeth undergoing early extraction usually show pathological signs of root resorption and are unsuitable for research ^8^. A challenge in obtaining a standardized sample for laboratory studies. An alternative for this is the use of prototyped resin models for the studies in primary teeth ^9^, ^10^. Prototype models in addition to enabling a better standardization of the research sample ^(^ ^11^, allow the possibility of countless replicates of the root complexity, for training different protocols ^12^.

Thus, this study aimed to evaluate the shaping ability and preparation time using a pediatric motor-driven rotary instrumentation compared to conventional motor-driven rotary instrumentation and manual instrumentation in resin prototypes of primary molars. The null hypothesis tested is that rotary instruments for pediatric use present similar time and shaping ability in the root canal preparation of prototype primary mandibular molars.

## Material and methods

Sample size calculation was undertaken using data from a previous study by Barasuol et al. ^13^ using G*Power (v3.1.9.2, University of São Paulo, Brazil), resulting in 17 canals per group, and to account for possible losses, 20 canals per group were used. Commercially available resin second mandibular deciduous molar prototypes (Denarte, São Paulo, SP, Brazil) were applied, using as sample (N=20) the distobuccal and distolingual root canals. They were randomly divided into three groups: Group 1 (G1): K files (Dentsply Maillefer, Ballaigues, Switzerland); Group 2 (G2): Sequence Baby File (MK-life Medical and Dental Products, Porto Alegre, RS, Brazil); Group 3 (G3): Sequence Rotary File (MK-life Medical and Dental Products, Porto Alegre, RS, Brazil).

### Micro-CT scanning

**Figure 1:**
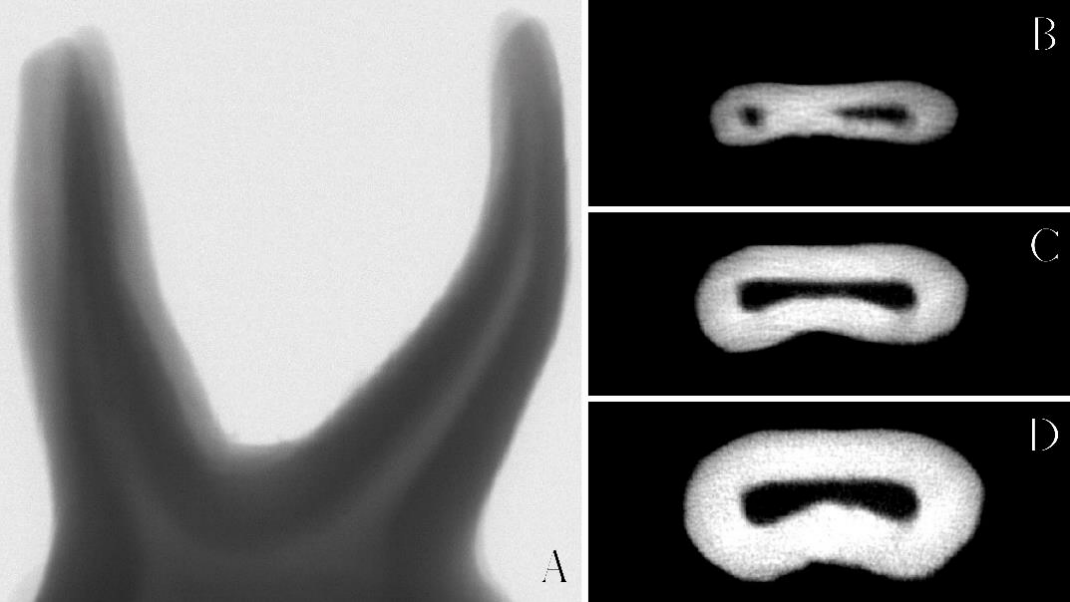
Microcomputed tomography scanned full image of primary molar (A) demonstrating three-dimensional reconstruction and the superimposed of the 3D thirds slices reconstruction, used for the measurements analyses according to the root thirds: apical (B), middle (C), and cervical (D).

Before and after instrumentation, the resin prototypes were micro-CT scanned (MicroCT Scanner, SkyScan 1174v2; Bruker-microCT, Kontich, Belgium), with the following acquisition parameters: 19.7 μm pixel size, 50 kV, 800 mA, rotation step of 1.0º and 360° rotation around the vertical axis (Figure 1).

### Root canal preparation and irrigation

One single operator performed all root canal preparation and irrigation procedures. The root canal was assessed by a using round Diamond bur (FG 1014; KG Sorensen, Cotia, Brazil) and the removal of the entire pulp chamber roof with an inactive top conical diamond tip (FG 3082; KG Sorensen, Cotia, Brazil). The working length (WL) was determined with a size #10 K file placed 2.0 mm shorter from the apex foramen. Next, the canals were prepared according to each study group:

G1: Root canal preparation was performed with K files (Dentsply Maillefer, Ballaigues, Switzerland), through crown-down technique employing balanced force for mechanical preparation of the root canals; The preparation started with a #15 K-file and reducing the diameter until it reached the working length. After reaching the working length, the root canal was enlarged in this extension to a diameter of 35.
G2: Root canal preparation was performed with pediatric motor-driven rotary instruments (Sequence Baby File; MK-life Medical and Dental Products, Porto Alegre, RS, Brazil) at continuous rotation, starting sequentially from instrument 17.08 (cervical third), 20.04, 25.04, and 30.04 (all WL) with an endodontic motor (Endus Duo Gnatus, Gnatus, São Paulo, SP, Brazil) set at 1.5N/cm torque and 375 rpm.
G3: Root canal preparation was performed with conventional motor-driven rotary instruments (Sequence Rotary File, MK-life Medical and Dental Products, Porto Alegre, RS, Brazil) at continuous rotation, starting sequentially from instrument 15.04, 20.06, 25.06 (WL), and 35.04 (2/3 WL) with an endodontic motor (Endus Duo Gnatus, Gnatus, São Paulo, SP, Brazil) set at 2N/cm torque and 425 rpm.

In all groups, irrigation was executed with 2 ml of saline at every instrument change. All prototypes were placed inside a customized device so that the instrumentation procedure was performed at the same working position.

During all preparation procedures, starting after the canal negotiation, the time was record continuously, without pausing during irrigations, with the aid of a stopwatch. The time registration started from the first instrument of each group until the last one, including the irrigation time. The number of samples, resulting in a mean instrumentation time, multiplied by the recorded time.

### Image comparison analyses

Both before and after, the scanned images were reconstructed three-dimensionally with NRecon ® software (v1.6.9, Bruker, Kontich, Belgium) and superimposed with the aid of DataViewer® software (v1.5.1.2, Bruker, Kontich, Belgium). CTAn ® software (v1.12, Bruker, Kontich, Belgium) was used to obtain the root canal deviation measurements and resin remnants at cervical, middle, and apical thirds. The measurements were repeated by one examiner (intraexaminer Kappa=0.86).

Root canal deviation was determined by the smallest distance between the tooth and the root canal wall on the mesial and distal sides, before instrumentation. Then, this was compared to the measurements of the same points after the instrumentation, according to the formula of a previous study (14): (X1-X2) - (Y1-Y2), where: X1 is the smallest distance between a point in the outer side of the root curvature and a point in the non-instrumented root canal wall at mesial direction; X2 is the smallest distance between a point in the outer side of the root curvature and a point in the instrumented root canal wall at mesial direction; Y1 is the smallest distance between a point in the outer side of the root curvature and a point in the non-instrumented root canal wall at distal direction; and Y2 is the smallest distance between a point on the outer side of the root curvature and a point in the instrumented root canal wall at mesial direction. The result equals zero means no deviations; positive value means deviation towards mesial; and negative value means deviation towards distal.

**Figure 2:**
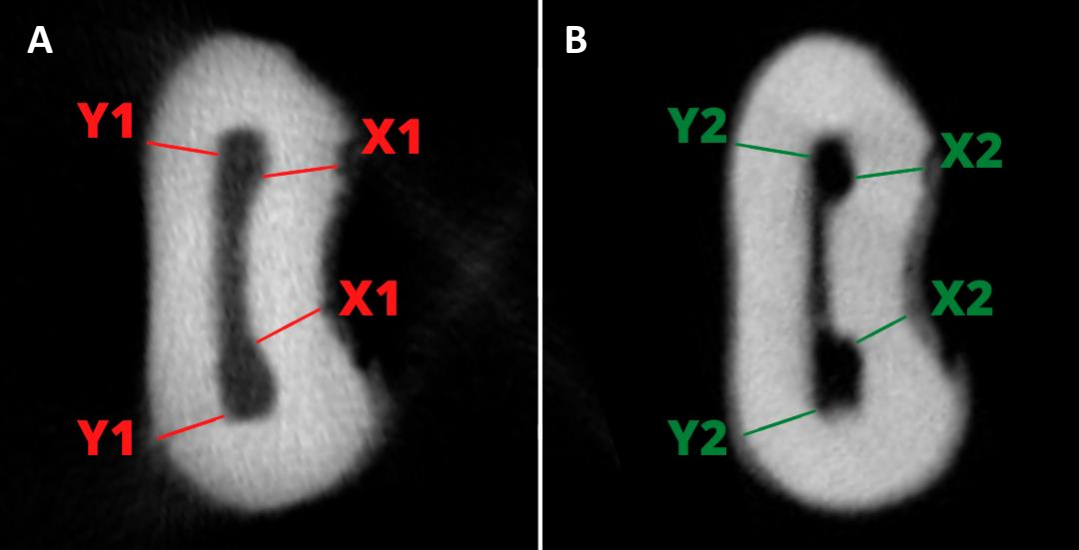
Cross-sectioned images showing distal canal in the middle third before (A) and after (B) preparation with SBF. Canal deviation and the remnant resin thickness were determined by measuring mesial (X1) and distal (Y1) before preparation, and mesial (X2) and distal (Y2) after preparation from the walls of the root canal.

The remnant resin thickness was calculated in percentages (%) at the three root thirds, by the formula (X1-X2) or (Y1-Y2), multiplied by por 100 and divided by either X1 or Y1, according to the side (mesial [X] or distal [Y]) ^(^ ^13^ (Figure 2).

### Statistical analysis

Root canal deviation, the percentage of remnant resin, and the instrumentation time were compared by ANOVA followed by the Tukey test, considering a normal distribution determined by the Kolmorov-Smirnov test. All tests adopted a level of significance of 5%.

RESULTS

Thirty prototype roots composed the sample. Due to the apical bifurcation, each group had 20 prototype root canals. No statistical significance difference occurred between groups regarding canal deviation (p=0.478944). The intergroup comparison between thirds showed statistically significant differences (p=0.000001) (Table 1). The cervical and apical thirds exhibited a deviation towards the distal side and the middle third towards the mesial side. There is no significant interaction between the factors group vs. root third.

**Table 1:** Mean and standard deviation (SD) of root canal deviation at cervical, middle, and apical root thirds (two-way ANOVA; Tukey test).

	Cervical mean ± SD (mm) [n=10]	Middle mean ± SD (mm) [n=10]	Apical mean ± SD (mm) [n=20]
K file	-1.074 ± 0.721 ª	0.478 ± 0.436 ᵇ	-0.132 ± 0.445 ᶜ
SBF	-0.447 ± 1.667 ª	0.754 ± 0.516 ᵇ	-0.337 ± 0.662 ᶜ
SRF	-0.796 ± 2.420 ª	0.979 ± 1.320 ᵇ	-0.086 ± 0.459 ᶜ

Different lowercase letters in the same line means statistically significant differences (p<0.05). Positive numbers mean deviation towards the mesial; negative numbers mean deviation towards the distal side.

The resin remnant percentage showed statistically significant differences between groups (p=0.000186), but not between thirds (Table 2). The comparison between the motor-driven rotary instrumentation (SBF and SRF) showed no statistically significance differences, but they promote statistically higher weariness than manual files (p<0.001). Motor-driven rotary instruments (SBF and SRF) had shorter working time than manual instruments (p<0.001) (Table 3).

**Table 2:** Mean and standard deviation (SD) of resin remnant percentage (%) after the instrumentation at cervical, middle, and apical root thirds.

	Cervical mean ± SD (%) [n=10]	Middle mean ± SD (%) [n=10]	Apical mean ± SD (%) [n=20]
K file	0.878 ± 0.039 ª	0.907 ± 0.052 ª	0.868 ± 0.126 ª
SBF	0.821 ± 0.036 ᵇ	0.850 ± 0.042 ᵇ	0.765 ± 0.156 ᵇ
SRF	0.761 ± 0.075 ᵇ	0.762 ± 0.082 ᵇ	0.809 ± 0.135 ᵇ

Different lowercase letters in the same column means statistically significant differences (p<0.05).

**Table 3:** Mean and standard deviation (SD) of instrumentation time.

	Working time mean ± DP (min)
K file	8.585 ± 1.233ª
SBF	3.91 ± 0.411ᵇ
SRF	4.69 ± 0.656 ᵇ

## Discussion

 This study evaluated the root canal preparation and working time between three endodontic instruments in the distal canal of primary molar prototypes through the measurement of canal deviation and the amount of resin remnant on micro-CT images. The null hypothesis was rejected meanwhile occurred differences between the rotary and manual instrumentation in the time and safety in the preparation of prototype inferior molars.

Micro-CT scanning is a noninvasive technique largely used in in-vitro studies because it enables a quantitative evaluation of the changes in internal anatomic structures of root canals ^(^ ^15^. Moreover, micro-CT scanning allows a three-dimensional view of tooth anatomy, with high accuracy, and promotes the understanding of the root canal system complexity influence on the endodontic treatment ^(^ ^16^,^17^.

Fast prototype models, made of resins that mimic the functional and radiological characteristics of dentin and used in others research centers ^11^, have been playing an important role in training and standardizing the sample for studies on endodontic treatments. These prototypes simplify the evaluation of different techniques and systems of rotary instruments and the capacity of root canal shaping ^(^ ^11^,^12^. In vitro studies improve the instrumentation and the decision-making process during clinical practice by providing further information ^(^ ^9^,^18^. In addition, standardization in research is a key factor because it enables the group comparison under similar morphological conditions ^(^ ^12^. However, despite the advantages described, the difference in hardness between human dentin and resin presents a limitation in the use of resin prototypes. Even with great advances in the area, until to date, no material has been able to simulate human dentin ^19^.

Canal deviation and resin remnant indicates the area, amount, and direction of wearing on root walls. These are important factors during the root canal instrumentation of primary teeth. Primary roots have more curved roots, with thinner dentin walls, and pulp chamber different of permanent tooth ^(^ ^5^,^20^. In addition, the rhizolysis process changes the internal and external topography according to the resorption degree ^(^ ^20^. Canal deviation verified by deviation did not show statistical differences between groups, that is, all instrumentation systems had similar weariness at the three thirds ^(^ ^21^,^22^. We expected that K-file exhibited the smallest deviation. Probably, this did not occur because despite of the smallest taper, K-file is composed of stainless steel, which cause less file deflection in curvatures. The small deflection results in greater weariness, similar to that of the rotary instruments. The intergroup comparison of the root thirds showed statistical significant differences, corroborating with Shaikh and Goswami ^22^. These authors compared the cleaning and shaping effectiveness of rotary, sonic, and conventional instruments in primary root canals and found greater deviations at cervical third, followed by middle and apical thirds.

The excessive removal of root structure may weaken the tooth ^(^ ^23^. Although the literature reaches no consensus on the amount of dentin to be removed to achieve the best root canal disinfection, some studies based on primary teeth report a greater removal of dentin with rotary instruments ^(^ ^21^,^23^. This agrees with the results of this present study, in which the rotary instruments showed significant greater weariness. Considering the analysis of the cervical and middle thirds, we observe that SRF instruments (taper 0.06) had greater values than SBF instruments (taper 0.04), with statistical similarity. These results may suggest that small taper of rotary instruments would be more indicated for the endodontic preparation of primary teeth ^(^ ^3^. Manual files were used in this study as control group because they are largely employed in the endodontic treatment of primary teeth ^(^ ^16^,^21^.

Motor-driven instruments had shorter working time than manual instruments, corroborating the literature ^(^ ^10^,^13^,^24^. This result is very important for the treatment of children because shorter treatments increase the child cooperation, decreasing a potential tiredness ^(^ ^25^. The working time comparison between the two rotary systems showed no statistically significant differences.

In childcare, reduced working time has important clinical relevance. It is crucial in relation to child anxiety, and operator and patient fatigue. Reduced instrumentation time enables optimized and more comfortable treatment.

To date, there is a few researches in the literature with the main focus in mechanized endodontics, but none in instruments developed specifically for deciduous teeth and among them, none evaluating canal deflection and amount of resin remaining.

The results of this in vitro study showed that motor-driven rotary instruments are suitable for the endodontic treatment of primary teeth, with useful advantages, as reported by the review of George et al. ^26^, who recommend the use of rotary instruments for this purpose. However, considering the complex root canal morphology of primary teeth and the importance of the cleaning and shaping for endodontic treatment success, further studies are necessary on instrumentation protocols with appropriate instruments. Moreover, further clinical trials are necessary to determine the reliability of these instruments under the clinical scenario, enabling a proper evaluation in all root canal systems.

The tested systems presented deviation and weariness, with similar results, and then the Sequence baby File could be considered suitable for mandibular primary molars.
